# Puberty, not age, marks the start of adolescent brain structural remodeling

**DOI:** 10.1016/j.dcn.2026.101835

**Published:** 2026-10-06

**Authors:** Jordan A. Chad, Olivia C. Klukas, Mohammad Ghasoub, Catherine Lebel

**Affiliations:** a Rotman Research Institute, Baycrest Academy for Research and Education, Toronto, Canada; b Department of Radiology, University of Calgary, Calgary, Canada; c Alberta Children’s Hospital Research Institute, Calgary, Canada; d Hotchkiss Brain Institute, Calgary, Canada

**Keywords:** Puberty, Adolescence, Brain structure, Cortical surface area, Subcortical volume, MRI

## Abstract

Many studies have shown that pubertal changes are associated with brain changes, but there lacks a clear understanding of what happens to the brain at puberty, and whether it opens the brain structural remodeling that characterizes adolescence. In this study, we used three longitudinal waves of structural MRI data from thousands of participants of the ABCD study aged 9–14 years to compare brain changes between age-matched children who had either reached or not reached a major pubertal milestone: menarche in females and fonarche (voice drop) in males. We found that only the children who had reached the milestone exhibited widespread contraction of cortical surface area and subcortical volume. The contraction was more pronounced in females, in line with previous evidence of how gonadal hormones regulate neuronal fine-tuning. Our findings suggest that reaching puberty, rather than reaching a particular age, begins the adolescent phase of structural brain development.

## 1 Introduction

There is no shortage of texts explaining how the body changes at puberty as it attains the mechanical requirements for reproduction, but it remains remarkably elusive how the brain changes at puberty as it attains the mental requirements for reproduction. Historically, male singers who had their vocal ranges preserved via castration before fonarche (pubertal voice drop) were reputed to lack the same independent self-regulation as post-fonarcheal men (Barbier, 1996). Scientific studies corroborate that adolescents and young adults who are hypogonadal tend to perform worse on cognitive tests than their post-gonadarcheal peers (Jordan et al., 2023, Lašaitė et al., 2014). It is therefore surprising that scientific research has consistently failed to find a clear link between pubertal status and executive functioning (for a systematic review, see (França et al., 2025)). Many studies have explored how the brain changes at puberty by testing for age-controlled correlations between structural neuroimaging markers and pubertal status measures (e.g., hormones, stage, timing, tempo), but have yielded inconsistent results (for reviews, see (Delevich et al., 2021; Goddings et al., 2019; Piekarski et al., 2023; Vijayakumar et al., 2018)). The typical conclusion of these studies is that children who are more pubertally developed exhibit more mature brain features than their age-matched peers (with conflicting reports on *which* brain features), but chronological age is the main determinant of adolescent brain structure. However, such data-driven approaches overlook the fact that proximate hormone effects in the brain do not immediately render long-term structural change (Heller et al., 2025), necessitating a priori hypotheses about how puberty modulates ongoing structural plasticity. Animal studies indicate that, rather than marking immediate adult-level neuromaturation, puberty marks an organizational window of neurodevelopment that proceeds over the course of adolescence (for reviews, (Romeo, 2003; Schulz et al., 2009; Schulz and Sisk, 2006; Sisk and Zehr, 2005)). Thus, in our present neuroimaging study, we tested specific hypotheses about how the event of puberty opens the adolescent phase of human brain structural development.

The first hypothesis was that puberty marks the start of cortical surface area contraction, an indicator of the cortical fine-tuning that hallmarks adolescence (Tamnes et al., 2017). An initial period of cortical fine-tuning occurs during the perinatal hormone surge, when overproliferated neurons are selectively pruned (Cowan et al., 1984). The process is modulated by estrogen receptor (ER) subtype in rodents, where ERβ binding promotes pruning while ERα binding mitigates it (Nilsen et al., 2000), such that aromatized androgens bind ERα to subdue pruning in males relative to females (Gegenhuber et al., 2022). In humans, the perinatal pruning is subdued and counteracted by androgen receptor (AR) binding, which likewise renders males with greater average cortical surface area than females (Alex et al., 2022). During adolescence, a second period of neuronal pruning occurs in rodents, yet again subdued in males relative to females due to gonadal hormones (Juraska and Drzewiecki, 2020). This adolescent pruning period has been gleaned in human neuroimaging, in that cortical surface area stops expanding and begins contracting around the time of puberty (Wierenga et al., 2014) (Fig. 1**A**). The extent of cortical surface area contraction in humans is associated with pubertal development even when controlling for age, especially in females (Beck et al., 2023, Curtis et al., 2024, McCann et al., 2026). These clues led us to hypothesize that the event of puberty opens an adolescent phase of cortical fine-tuning marked by surface area contraction, and that females exhibit more of this contraction than males.

**Fig. 1:**
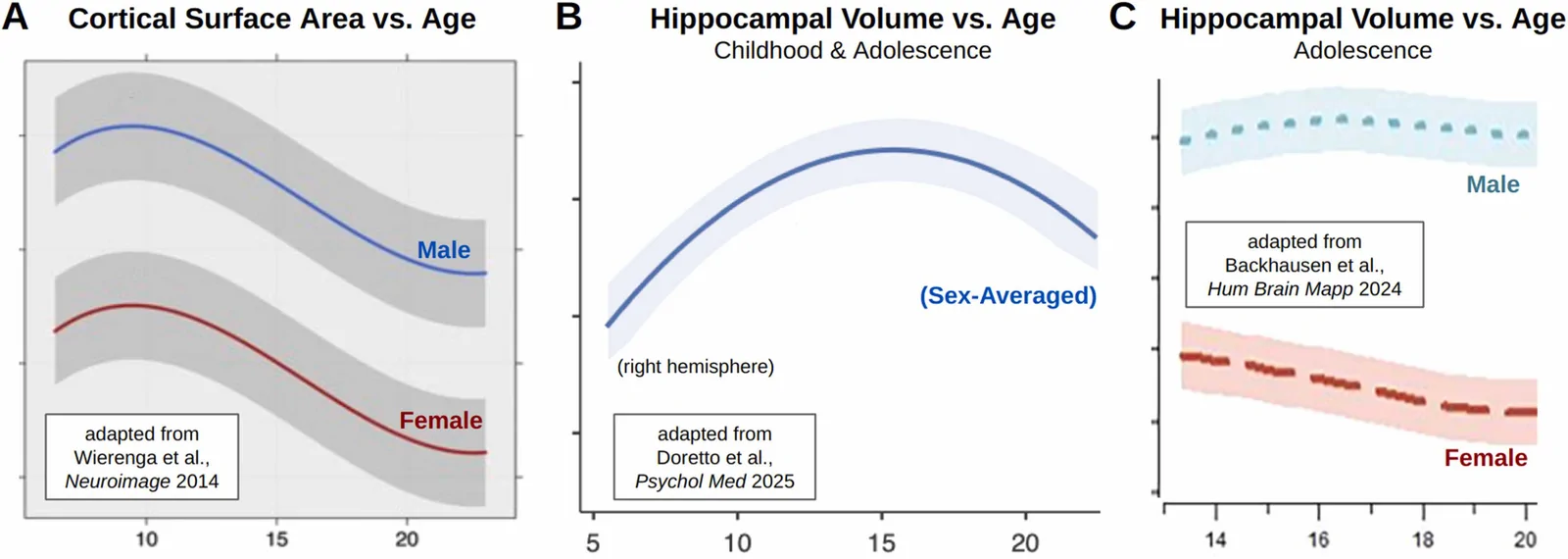
**Does puberty mark a turning point in structural brain development?** (A) Cortical surface area stops expanding and begins contracting around the time of puberty in both sexes (Wierenga et al., 2014). (B) Subcortical structures like the hippocampus exhibit analogous expansion-contraction trajectories in development, but subcortical contraction appears to begin later than puberty (Doretto et al., 2025). (C) When broken down by sex, females seem to begin subcortical contraction as early as puberty, but males undergo continued post-pubertal expansion before entering contraction (Backhausen et al., 2024). Note that the axes are not the same scale across panels.

Our second hypothesis was that puberty also marks the start of *subcortical* tuning, though not necessarily the immediate start of its structural contraction, since subcortical structures are still growing in early adolescence (Fig. 1**B**). This hypothesis was based on the subcortex’s dense array of sex hormone receptors (Cara et al., 2021, Osterlund and Hurd, 2001), which tune dispositions to mature experiences that drive the cortical contraction trajectories (Panksepp, 2011; cf. Larsen and Luna, 2018). In the language of the expansion-renormalization model of plasticity (Wenger et al., 2017), ARs promote sustained expansion of the subcortex (Kuwahara et al., 2021) while ERs balance its level of renormalization, with ERβ boosting fine-tuning and ERα mitigating it (Matthews and Gustafsson, 2003). We hypothesized that male subcortical structures undergo a pubertal growth spurt before entering their contraction trajectories due to ERα and AR binding, whereas female subcortical structures begin contracting right during puberty due to ERβ binding (Fig. 1**C**). We further maintained specific sub-hypotheses about how subcortical volumes change at puberty, based on their relative hormone receptor densities and previous neuroimaging research (Box 1).

Box 1 **Sub-Hypotheses About Subcortical Volume Changes**

**Amygdala.** The amygdala and hypothalamus have the highest densities of ERα and AR receptors in the brain (Cara et al., 2021, Osterlund et al., 2000). Animal research shows that puberty renders net amygdala contraction in both sexes over the course of adolescence (Nuruddin et al., 2013). However, androgens initially cause continued growth and suppressed contraction of the male amygdala in early adolescence, yielding more aggressive and less anxious social behaviors in male than female animals (Ahmed et al., 2008, Cooke and Woolley, 2009, Oliveira et al., 2024, Sano et al., 2016, Tsukahara et al., 2011). In human functional neuroimaging studies, matured amygdala activation during social face processing is the most consistent hallmark of reaching puberty in both sexes (for a meta-analysis, see (Dai and Scherf, 2019)), and the male amygdala briefly continues to grow following puberty, while the female amygdala begins to contract (Backhausen et al., 2024, Carrick et al., 2025, Goddings et al., 2014). The animal studies did not examine laterality, but human neuroimaging has found that only the right amygdala is associated with certain pubertal measures (Bramen et al., 2011, Campbell et al., 2022, Ferri et al., 2014, Herting et al., 2014, Vijayakumar et al., 2021). Testosterone appears to mediate male social aggression via activity in the right amygdala specifically (Radke et al., 2015), which adolescent boys activate more than adolescent girls during face processing (Schneider et al., 2011) due (at least in part) to testosterone (Beking et al., 2020). We therefore hypothesized that puberty marks a growth spurt in the male amygdala that is possibly localized to the right hemisphere, alongside a growth “stunt” in females.  

**Hippocampus.** The hippocampus has the highest density of ERβ receptors in the brain (Warfvinge et al., 2020). We hence hypothesized that puberty marks the start of sharp hippocampal contraction in females, as has been observed in mice (Afroz et al., 2016) and human feminizing hormone therapy (Seiger et al., 2016). Like the cortex, the hippocampus has a relatively low ERα:ERβ ratio (González et al., 2007), so we hypothesized a less pronounced male pubertal growth spurt in the hippocampus compared to the amygdala, as has been previously evidenced in humans (Backhausen et al., 2024). Human studies have nonetheless found continued male post-pubertal hippocampal growth (Backhausen et al., 2024, Carrick et al., 2025, Goddings et al., 2014), consistent with ARs promoting ongoing hippocampal growth (Blankers and Galea, 2021, Kight and McCarthy, 2020). These hormonal effects would explain why, during spatial navigation, men tend to rely on geometric cues by engaging large hippocampi while women tend to rely on visual landmarks by engaging more finely-tuned hippocampi and cortex (Yagi and Galea, 2019). We thus specifically hypothesized that puberty marks the start of hippocampal contraction in females alongside sustained hippocampal growth in males.  

**Hypothalamus.** Drives instantiated in the hypothalamus (e.g., sex and sleep) tend to be tuned by ERα rather than ERβ activation (Le Moëne et al., 2019, Tetel and Pfaff, 2010, Torres et al., 2024), which is further pronounced by AR activation (Karatsoreos et al., 2007, Sartorius et al., 2014). Accordingly, the hypothalamus’ medial preoptic area undergoes less neuronal pruning in adolescent males than females (Swaab and Hofman, 1988) which has been attributed to testosterone (Dodson and Gorski, 1993). Neuroimaging studies find that adult males have larger nuclei than adult females throughout the hypothalamus (Huang et al., 2026). Hypothalamic trajectories have not been well-studied in neuroimaging, but based on the large nuclei in adult males and high ERα:ERβ ratio and AR density, we hypothesized that puberty marks a growth spurt of the male hypothalamus. In females, since the hypothalamus is sparse in ERβ receptors compared to other subcortical structures (Osterlund and Hurd, 2001), we anticipated less contraction of the hypothalamus compared to the other structures.  

**Thalamus.** The thalamus’ paraventricular nucleus (PVN), which relays flagged stimuli into neighboring subcortical structures (Penzo and Gao, 2021), is abundant in ERβ but not ERα (Fan et al., 2006, Mitra et al., 2003). Estrogens tune PVN activity in female rats (Hilz et al., 2019, Krajnak et al., 2003, Ueyama et al., 2006), which is linked to females being more risk-averse than males (Ishii et al., 2018). In human females but not males, the thalamus begins to contract in early adolescence (Backhausen et al., 2024, Carrick et al., 2025). We therefore hypothesized that puberty marks the start of thalamic contraction in females, more sharply than in the ERβ-sparser amygdala and hypothalamus, but less sharply than in the ERβ-richer hippocampus. In males, since the thalamus is less dense in ARs than the other structures (Cara et al., 2021) and grows less after puberty (Carrick et al., 2025), we did not hypothesize much prolonged post-pubertal male thalamus growth.

To test whether puberty marks these discrete turning points in brain development, we needed to operationalize puberty with a discrete event. This task is known to be challenging (Beltz et al., 2025, Goddings, 2024). A common method of measuring puberty is to sum categorical measures of somatic development (e.g., reports on changes in height, hair, skin, etc.) into a composite continuous score, such as the pubertal development scale (PDS) (Petersen et al., 1988). For our purposes, however, we required binary measures that are direct and inevitable culminations of puberty, as opposed to indefinite processes like body growth and skin changes that are heavily influenced by non-pubertal factors. Thus, for females, we chose their most discrete pubertal milestone: whether they had reached their first menstrual period (menarche), an indicator of whether there are sufficient female hormones to stimulate uterine thickening. For males, we chose a parallel pubertal milestone: whether they had reached their first clear sign of voice drop (fonarche), an indicator of whether there are sufficient male hormones to stimulate larynx growth. The respective timings of menarche and fonarche within pubertal development are comparable (Hodges-Simeon et al., 2013), and both are associated with the same genetic etiology (Day et al., 2015) and similar growth spurts in height (Hägg and Taranger, 1980). Menarche further corresponds to a subtle female voice drop (Bugdol et al., 2018), reiterating that fonarche is an appropriate male counterpart measure to menarche.

In this study, we leveraged longitudinal data from participants of the Adolescent Brain Cognitive Development (ABCD) between ages 9–14 years to test that only the children who have reached menarche or fonarche undergo the hypothesized structural changes. This study thus sought evidence that the characteristic age-related changes in the adolescent brain are initiated at puberty rather than a particular age.

## 2 Methods

### 2.1 Data sources

All data from the current study were obtained from participants of the ABCD Study via Data Release 5.0. The study recruited over 10,000 children aged 9 or 10 years from 21 sites across the United States in 2017–2018 (with child assent and caregiver consent) to be followed with annual pubertal development assessments and biennial MRI brain scans over the course of 10 years. Detailed information on the recruitment process (Garavan et al., 2018), as well as the MRI data acquisition (Casey et al., 2018) and analysis (Hagler et al., 2019), can be found elsewhere. The three longitudinal MRI timepoints in the current data release are:

Year 1 (2017–2018) = Age 9 or 10 years

Year 3 (2019–2020) = Age 11 or 12 years

Year 5 (2021–2022) = Age 13 or 14 years

We obtained measures of cortical surface area from the spreadsheet mri_y_smr_area_dsk.csv, which measures the area of 34 cortical parcellations per hemisphere via the Desikan-Killiany atlas (Desikan et al., 2006). We obtained measures of subcortical volume from the spreadsheet mri_y_smr_vol_aseg.csv, which are computed based on (Fischl et al., 2002). We obtained pubertal measures from the spreadsheet ph_p_pds.csv, described below.

### 2.2 Pubertal measures

The ABCD study administered annual questions about pubertal development to children and their parents/caregivers, and we used the parent/caregiver responses in this study as per the recommendation (Cheng et al., 2021). We assessed female pubertal development at each survey via the answers (yes or no) to: “Have you/she begun to menstruate (started to have your period)?” We assessed male pubertal development using: “Have you noticed a deepening of your/your child’s voice?”, which offered four response options:

a) not yet started
b) has barely started
c) is definitely underway
d) seems complete

We defined pre-fonarche as answering (a) or (b), and post-fonarche as answering (c) or (d). Among the 11,868 participants included in the data release, we excluded 56 female participants and 210 male participants from our analyses because their reported menarcheal or fonarcheal status exhibited regressions over time. We also excluded an additional 2 participants who reported menarche status at one timepoint and fonarche status at another. Note that we classified participants as female or male based on whether they reported menarche or fonarche status, rather than based on their reported sex per se. In cases of missing reports, participants were classified as pre-milestone if a later report reported them as pre-milestone, and post-milestone if an earlier report reported them as post-milestone. In cases where missing pubertal status could not be logically inferred, participants with missing data were removed from analyses involving that timepoint.

### 2.3 Age-matching

We ran a series of analyses comparing change from Year X to Year Y between pubertally-defined groups, as described below. Participants assessed at a given Year were not necessarily all the exact same age (as per “interview age” in the spreadsheet abcd_y_lt.csv), and more pubertally-developed participants were on average slightly older than their less pubertally-developed peers. We therefore needed to match the groups for average age and duration between assessments. These matchings were done using the optimal full-matching algorithm (Hansen and Klopfer, 2006) implemented in the MatchIt R package (Ho et al., 2011). Optimal full-matching allowed us to retain maximum group sizes, unlike optimal pair matching, which would have required discarding data from the larger group to match the size of the smaller group. We modeled advanced pubertal development at a given Year as a treatment on the average age; i.e., we defined the more pubertally-developed group as the treatment group, and the less pubertally-developed group as the control group. The algorithm then applied weighting to each participant in a specified group so that its weighted average age matched the average age of the other group, thereby estimating the average treatment effect (Hansen and Klopfer, 2006).

To decide which group to specify for weighting, we applied the following rules to maximize sensitivity to change with age amid turning points between growth and contraction: For testing hypotheses about surface area or volume *contraction*, which should be more pronounced at older than younger ages, we applied weighting to each participant in the less developed (control) group so that its weighted-average age (and inter-assessment duration) matched that of the more developed (treatment) group (i.e., we measured the average treatment effect on the treated, ATT). For testing hypotheses about ongoing volume *growth*, which should be more pronounced at younger than older ages, we applied weighting to each participant in the more developed (treatment) group so that the its weighted-average age (and inter-assessment duration) matched that of the less developed (control) group (i.e., we measured the average treatment effect on the controls, ATC). The specific analyses are described below. Weighted-average ages and inter-assessment durations between age-matched groups never significantly differed at p < 0.05 as per a Welch’s *t*-test.

### 2.4 Analyses

Our first analyses pertained to changes from Year 3 to Year 5, since there were large numbers of children who were pre-milestone throughout this duration as well as children who were post-milestone throughout this duration. (There were very few children who reached their milestone by Year 1, so examining post-milestone changes beginning from Year 1 would have had little statistical power.) We calculated change from Year 3 to Year 5 in (i) surface area in each of the 34 cortical regions (per hemisphere), and (ii) volume of each of the four subcortical regions (per hemisphere), for the following four groups:

a. females who reached menarche by Year 3 (n = 450)
b. males who reached fonarche by Year 3 (n = 170)
c. females who did not reach menarche by Year 5 (n = 100)
d. males who did not reach fonarche by Year 5 (n = 406)

The statistical significance of each change was determined by a weighted paired *t*-test. The group that had not reached menarche/fonarche by Year 5 was weighted to match the group that had reached menarche/fonarche by Year 3 in terms of (i) age at Year 3 and (ii) duration between Year 3 to Year 5. For the cortical surface area analysis, we then:

1. normalized each raw surface area change by the mean baseline surface area;
2. output lists of relative surface area changes per region into text files;
3. converted these text files into FreeSurfer Parcellation Statistics files using mri_convert with the “parcstats” option;
4. visually overlaid these parcstats files on FreeSurfer's fsaverage pial surface.

For the subcortical volume analyses, in addition to studying the effect of reaching the milestone before versus after the change, we were additionally interested in studying the effect of reaching the milestone *during* the change (since this was expected to modulate ongoing subcortical growth; see Box 1). We therefore used data at all three timepoints to conduct weighted Welch’s *t*-tests pertaining to:

a. two-year volume change from Year 1 to Year 3 among females who were pre-menarche at Year 1, comparing those who reached menarche by Year 3 (n = 1312) versus those who did not (n = 2196);
b. two-year volume change from Year 1 to Year 3 among males who were pre-fonarche at Year 1, comparing those who reached fonarche by Year 3 (n = 554) versus those who did not (n = 3563);
c. two-year volume change from Year 3 to Year 5 among females who were pre-menarche at Year 3, comparing those who reached menarche by Year 5 (n = 626) versus those who did not (n = 100);
d. two-year volume change from Year 3 to Year 5 among males who were pre-fonarche at Year 3, comparing those who reached fonarche by Year 5 (n = 784) versus those who did not (n = 406).

We conducted these tests for the amygdala, hippocampus, ventral diencephalon (which includes the hypothalamus), and thalamus proper (here called “thalamus”). The statistical significance of each individual change was determined by a weighted paired *t*-test. For group comparisons of the changes, the group that did not reach menarche was age-weighted to the group that did, and the group that reached fonarche was age-weighted to the group that did not (as described in the “Age-matching” subsection of Methods). Note that we conducted all *t*-tests on absolute volume changes rather than relative changes (i.e., without controlling for baseline volumes). This approach was chosen because average relative changes can obscure interpretations of directional effects (e.g., if negative changes have higher baseline volumes than positive changes). Instead, we separately compared the absolute volumes at baseline between groups using weighted Welch’s *t*-tests to confirm differences did not already exist before the pubertal milestone. We normalized each raw volume change by the mean baseline volume before displaying in descriptive tables. A summary of analyses is displayed in Table 1.

**Table 1:** **Study overview.** See the Methods text for more details.

**Hypothesis 1**. Cortical surface area contraction only after menarche and fonarche	
**Test**. Assess cortical surface area change from Year 3 to 5 among children who are:	
• pre-menarche at Year 3&5 (n = 100)	• post-menarche at Year 3&5 (n = 450)
• pre-fonarche at Year 3&5 (n = 406)	• post-fonarche at Year 3&5 (n = 170)
	
**Hypothesis 2A**. Subcortical volume contraction only after menarche (not fonarche)	
**Test**. Assess subcortical volume change from Year 3 to 5 among children who are:	
• pre-menarche at Year 3&5 (n = 100)	• post-menarche at Year 3&5 (n = 450)
• pre-fonarche at Year 3&5 (n = 406)	• post-fonarche at Year 3&5 (n = 170)
	
**Hypothesis 2B**. Subcortical growth lesser upon menarche and greater upon fonarche	
**Test 1**. Assess subcortical volume change from Year 1 to 3 among children who are:	
• pre-menarche at Year 1&3 (n = 2196)	• post-menarche btwn Year 1&3 (n = 1312)
• pre-fonarche at Year 1&3 (n = 3563)	• post-fonarche btwn Year 1&3 (n = 554)
	
**Test 2**. Assess subcortical volume change from Year 3 to 5 among children who are:	
• pre-menarche at Year 3&5 (n = 100)	• post-menarche btwn Year 3&5 (n = 626)
• pre-fonarche at Year 3&5 (n = 406)	• post-fonarche btwn Year 3&5 (n = 784)

The study included a total of 400 analyses. We present raw p-values for the results of each analysis and indicate if they fall below the Bonferroni threshold of p < 0.0001 (0.05/400). For visually displaying surface area changes across cortical regions, we did not want to spuriously support our hypothesis by missing real effects in pre-milestone children, so we thresholded by an effect size of 0.95%. This threshold corresponded to the smallest effect size that was significant at p < 0.0001 in n = 450 post-menarcheal children, ensuring that equally large effect sizes in n = 100 pre-menarcheal children did not get missed simply because the latter sample was smaller.

## 3 Results

Hypothesis Test 1 *Cortical surface area contraction after puberty*

Fig. 2 displays the weighted two-year average change in cortical surface area from Year 3 to Year 5 (age 12–14 y) among children who were either pre- or post-milestone for the entire duration of the change. As per the weighting, the pre- and post-milestone groups were not of significantly different age at baseline (p = 0.82 for females, p = 0.90 for males) nor did they undergo significantly different durations between assessments (p = 0.59 for females, p = 0.75 for males). Post-milestone children exhibited widespread cortical surface area contraction during the two years, while pre-milestone children did not exhibit much cortical surface area contraction during these same two years. Contraction was more widespread and of greater magnitude in post-menarcheal than post-fonarcheal children.

Hypothesis Test 2A *Subcortical contraction after menarche*

**Fig. 2:**
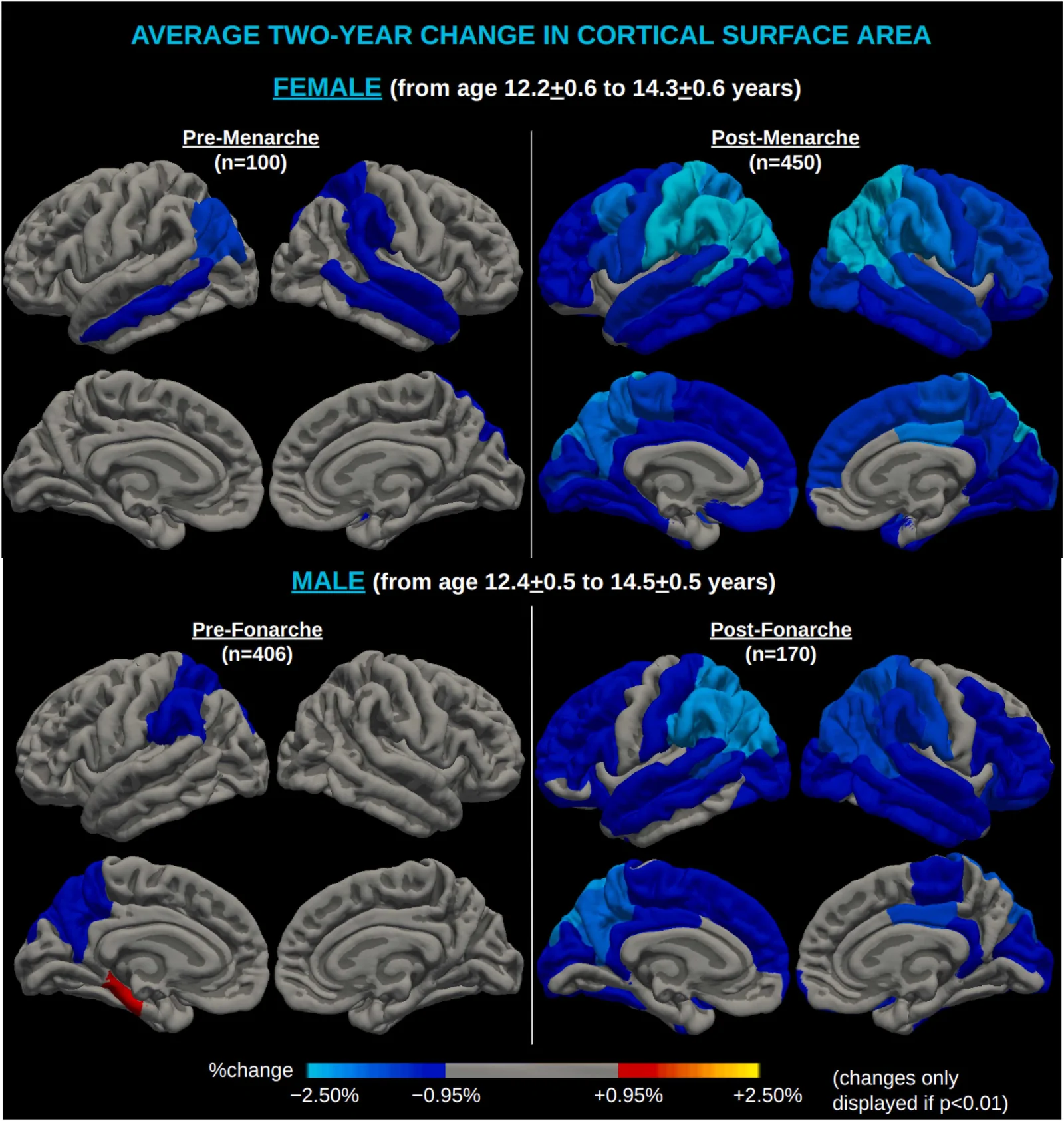
**Puberty, not age, marks the start of widespread cortical surface area contraction.** The cortex is parcellated into 34 regions per hemisphere, with each region color-coded by its average two-year change in surface area (normalized by average baseline surface area). Averages are weighted to enforce age matching, as described in text. Changes are not displayed (i.e., the region is gray) if smaller in magnitude than 0.95%, or if p> 0.01 as per a weighted one-sample *t*-test.

Table 2 displays the weighted two-year average change in subcortical volume among the same groups of children. Unlike cortical surface area, which had already stopped growing before the pubertal milestone (left panels of Fig. 2), subcortical volumes often continued to significantly increase right up to puberty. This growth was most apparent among the pre-fonarcheal males, who exhibited significant growth of the hippocampus and ventral diencephalon (p < 0.0001 bilaterally), as well as growth of the thalamus (p < 0.0005 for right, p < 0.005 for left) and amygdala (p < 0.05 bilaterally) that were not significant after Bonferroni correction. The pre-menarcheal females exhibited less growth of these structures, with no average growth at all in the amygdala, yet still no significant contraction. Trends were different among the children who reached their milestone. Post-milestone children exhibited less average growth and/or more average contraction than the pre-milestone children. Among males, this meant less average growth but still no significant contraction. Among females, this meant significant contraction and no more significant growth.

Hypothesis Test 2B *Subcortical growth stunt at menarche and growth spurt at fonarche*

**Table 2:** **Puberty, not age, marks the start of subcortical contraction in females, and decelerated subcortical growth in males.** The table displays average two-year change in volume (normalized by average baseline volume) in four subcortical structures per hemisphere. Change was always smaller (or more negative) in the post- versus pre-milestone group: In post-milestone females, change was generally negative (indicating contraction); in post-milestone males, change was less positive than in pre-milestone (indicating decelerated growth). Statistical significance p-values are indicated in brackets and emphasized via color fills, with warm colors indicating positive changes and cool colors indicating negative changes, and white indicating p> 0.1.

Table 3 displays the effect of reaching pubertal milestones between age 10–12 y, and Table 4 displays the effect of reaching pubertal milestones between age 12–14 y. In both cases, females who were pre-menarcheal at baseline and reached menarche two years later exhibited less two-year subcortical growth than the pre-menarcheal females who did not reach menarche two years later. This group difference was significant with Bonferroni correction in the hippocampus and thalamus (p < 0.0001). Note that menarche marked stunted growth in the bilateral thalamus irrespective of timing, but *earlier* menarche marked stunted growth in only the right hippocampus (Table 3), while *later* menarche marked stunted growth in only the left hippocampus (Table 4). Among males who were pre-fonarcheal at baseline, those who reached fonarche two years later exhibited *more* two-year subcortical growth than those who did not, especially in the right amygdala and bilateral ventral diencephalon. The right amygdala spurt was only evident for earlier fonarche (p < 0.005; Table 3) and not later fonarche (p > 0.1; Table 4).

**Table 3:** **Reaching menarche marks subcortical growth stunts and reaching fonarche marks subcortical growth spurts (age 10–12 y).** The table shows average two-year growth in subcortical volume among children who were pre-milestone at Year 1. Females who reached menarche by Year 3 exhibited *less* average growth than females who did not, while males who reached fonarche by Year 3 exhibited *more* average growth than males who did not. Statistical significance p-values are indicated in brackets and emphasized via color fills, with warm colors indicating positive changes/differences and cool colors indicating negative changes/differences, and white indicating p> 0.1. There were few average differences in volume between the groups at baseline (all p > 0.01 and most p > 0.1).

**Table 4:** **Reaching menarche marks subcortical growth stunts and reaching fonarche marks subcortical growth spurts (age 12–14 y).** The table shows average two-year growth in subcortical volume among children who were pre-milestone at Year 3. Females who reached menarche by Year 5 exhibited *less* average growth than females who did not, while males who reached fonarche by Year 5 exhibited *more* average growth than males who did not. Statistical significance p-values are indicated in brackets and emphasized via color fills, with warm colors indicating positive changes/differences and cool colors indicating negative changes/differences, and white indicating p> 0.1. There were few average differences in volume between the groups at baseline (all p > 0.01 and most p > 0.1).

## 4 Discussion

This work provides concrete evidence that children who have reached their pubertal milestone have also reached a milestone in their structural brain development. We found that both menarche and fonarche mark the start of broad cortical surface area contraction independent of age, more pronounced following menarche than fonarche. Subcortical structures expand right up to puberty (after cortical surface area reaches its peak), but reaching menarche appears to mark a *stunt* in subcortical growth and shifts it towards contraction, whereas reaching fonarche appears to mark a *spurt* in subcortical growth before decelerating it and shifting it towards contraction. These findings support the theory that puberty corresponds to a turning point in brain development not reached through age alone.

Scientists have long struggled to understand how puberty sets off apparent turmoil in brain development trajectories, given that children are often compelled to seek out more arousing and risky sensations after puberty (Steinberg et al., 2018) and psychiatric illness often does not emerge until after puberty (Paus et al., 2008). The conventional theory that the adolescent brain is simply adjusting to “raging hormones” lacks a solid scientific basis (Buchanan et al., 1992). Since adolescent neuroimaging markers are more strongly correlated with age than pubertal markers (Curtis et al., 2024), adolescent brain theories now often treat adolescence as a consequence of age without regard to puberty (e.g., (Casey et al., 2025)). Our present work suggests that, while structural changes indeed occur with age and not instantaneously at puberty, it is puberty that marks the *start of the adolescent phase* of age-related changes. This is of course not to say that puberty marks the start of all structural changes in the adolescent brain. Some degree of learning and neuroplasticity is ongoing regardless of puberty, and cortical thinning begins in early childhood before cortical surface area contraction at puberty (Wierenga et al., 2014). Gonadectomized and puberty-delayed animals still undergo brain structural changes during adolescence (Andersen et al., 2002, Boivin et al., 2018, Hoops et al., 2024). However, when puberty proceeds without disruption, the event of puberty significantly modulates these changes (Drzewiecki et al., 2016, Juraska and Willing, 2017), supporting the idea that puberty catalyzes the start of a new phase of learning—and its accompanying emotional turmoil—amid ongoing brain development. Future work will corroborate whether puberty marks the start of mood and behavioral processes that associate with the brain changes.

A key question is *how* puberty might start this phase. It has been previously argued that puberty triggers the adolescent phase of learning via estrogens binding cortical ERβ receptors on inhibitory interneurons to denoise and attune the cortex (Piekarski et al., 2017). However, this hypothesis does not explain why male puberty, which primarily involves androgenic ERα and AR binding, would trigger cortical surface area contraction. While estrogen and antiandrogen treatment in human adulthood decreases gray matter volume, androgen treatment *increases* it (Burke et al., 2018, Pol et al., 2006, Seiger et al., 2016, Zubiaurre-Elorza et al., 2014), which in the cortex is driven by cortical surface area *expansion* (Kilpatrick et al., 2019). Our study suggests that the natural androgen and estrogen changes following puberty have an analogous effect of mitigating and promoting structural contraction, but does not explain why the structural contraction begins in the first place. It is possible that pubertal onset is in part an effect (rather than cause) of the brain’s readiness for cortical contraction, such that energy formerly used by the brain can be allocated to the body (Kuzawa et al., 2014). That said, a very likely prompter of cortical tuning is *subcortical* tuning, which denoises affective dispositions towards new experiences that drive the course of cortical contraction ((Panksepp, 2011), cf. the “driven dual systems model of adolescence” (Luna and Wright, 2016)). According to this hypothesis—which we will expand upon in future work—new subcortical tendencies towards the complex social world initiate a phase of cortical fine-tuning as pubescents figure out how to navigate it.

The current study provides novel insight into the structural manifestations of the subcortical tuning. As hypothesized, we found that fonarche marked the largest growth spurts in the ERα-dense amygdala and ventral diencephalon (which includes the hypothalamus), while menarche marked the largest growth stunts in the ERβ-dense hippocampus and thalamus. However, some of our findings were not hypothesized. Males exhibited a right amygdala growth spurt upon early fonarche but not upon late fonarche (Table 3 vs. Table 4), apparently because the amygdala stopped growing altogether early in adolescence (Table 2). This earlier-than-expected leveling off of amygdala growth may be related to the contemporary rise of adolescent anxiety (Anderson et al., 2025) that may in turn be related to early amygdala contraction (Liu et al., 2024). We also did not explicitly hypothesize that early menarche would be associated with a *right* hippocampus growth stunt (Table 3) while late puberty is associated with a *left* hippocampus growth stunt (Table 4), though this is consistent with previous evidence that plastic growth of the right hippocampus closes in early adolescence slightly before the left reaches its peak (Uematsu et al., 2012). The timing of puberty is linked to long-term sex-specific psychosocial development (for a review, see (Graber, 2013)), and male puberty is generally later than female puberty (see the proportions of post-menarche versus post-fonarche at age 12–14 y in Fig. 2), so it may be worthwhile for future work to investigate whether the age at puberty relates to the distribution of structural changes.

We binarized puberty by whether children reached menarche or fonarche. Previous work using continuous (rather than discrete) puberty measures found that more advanced puberty is associated with less gray matter volume in females and more in males *at a given age* (Bramen et al., 2011, Peper et al., 2009), but here we were interested in whether puberty *opens a window* of less gray matter *growth* in females and more in males *with advancing age*. We thus tested binary hypotheses about pre- versus post-puberty rather than the temporal progression of pubertal development (cf. (McCann et al., 2026)). We investigated peripubertal timepoints in early adolescence and did not aim to reproduce the ensuing adolescent trajectories (Fig. 1); the latter task will need to be set aside until the remaining ABCD data are acquired and released. The current study was also not designed to deduce whether the hypothesized neurodevelopmental turning points occur (a) shortly before, (b) right at, or (c) shortly after the pubertal milestones. Pre-milestone participants were likely heterogenous in terms of pubertal status (e.g., some presumably already underwent breast/testicular enlargement while others did not), and it is possible that certain early pubertal events are associated with brain changes undetectable to the current study. For example, early female puberty may be associated with a subcortical growth spurt prior to the post-menarcheal fine-tuning. Our study merely established that the start of adolescent brain structural remodeling is associated with pubertal development over and above age.

We note that our measure of fonarche is inherently less reliable than menarche, since fonarche is less of a discernible or instant event than menarche. Parents could plausibly mistake fonarche for laryngitis or mistake laryngitis for fonarche (for example), so we binarized fonarche as a voice deepening that is “definitely underway”, and treated a voice deepening that has “barely started” as pre-fonarche. Our findings obtained using this fonarche definition are consistent with previous findings on male puberty using other measures, in that male puberty is less associated with structural brain contraction than female puberty. We chose fonarche because (a) its prevalence and timing within the sequence of pubertal development is comparable to menarche, and (b) it is the most discernible and binarizable male pubertal event that the ABCD study recorded. The most standard clinical marker of male puberty, testicular enlargement (not recorded by ABCD), is unlikely to be reliably reported by most parents or children, and it is an early pubertal event comparable to breast enlargement rather than menarche. Other proposed male pubertal milestones are similarly suboptimal. The timing of first ejaculation (also not recorded by ABCD) is partly influenced by non-endocrinological factors and it is unfeasible for parental reports (Chad, 2020). Measurement of sperm production (again not in ABCD), which can be binarized by recording whether or not sperm is present in urine samples, is plagued by false negatives (Jørgensen et al., 1991). In light of these challenges, our current study introduces fonarche as a useful and feasible male counterpart measure to menarche.

A main limitation of our study design was the sharp restriction of sample size to meet the given criterion (e.g., pre-menarche at Year 5, post-fonarche at Year 3, etc.), and the necessity to exclude participants who met the criterion but did not have data available at all relevant timepoints (to measure change). We chose not to further restrict our sample with additional filtering or outlier examination (beyond the ABCD study’s pre-implemented quality control), rendering further limitations. There was likely substantial variability in a multitude of factors across participants, which may have confounded inter-group comparisons (see the high p-values of the Welch’s *t*-tests in right columns in Tables 3 and 4). Moreover, data were collected across 21 sites and there may have been site-related variability. While the neuroimaging measures used in our study demonstrate high test-retest reliability across MRI scanners, sequences, and coils (Knussmann et al., 2022, Yan et al., 2020), it would be best practice to control for site differences in future ABCD analyses. We also did not control for mental and physical health or social factors at either the given or later timepoints. It is possible that a higher proportion of the early-puberty groups will go on to develop psychiatric illness than of the late-puberty groups, which may already influence early neuroimaging markers. It is also possible that the early-puberty groups were more biologically mature than the late-puberty groups in respects other than pubertal status, in which case matching groups by bone age (e.g., through wrist x-rays) could be more informative than chronological age. In any case, there is plenty more work to do to characterize the link between puberty and brain development, and this study merely sought to fill in a few important conceptual gaps.

As a final remark, we note that our study is the first to validate that the start of adolescent brain structural remodeling can be classified under the umbrella term “puberty”. Neuroimaging studies that employ broad composite measures of puberty are unable to definitively assert that their findings are temporally related to pubertal endocrinology, since these studies generally label children who lack acne or growth spurts as not fully pubertally developed despite these developments not being universal to healthy puberty (Henneberg et al., 2025, Li et al., 2025). To resolve this issue, we employed narrow endocrinological milestones that are indissociable from puberty so that we could definitively assert (assuming limited reporting errors) that our findings are temporally related to pubertal endocrinology. These milestones are more robust endocrinological measures than hormonal assays (Beltz et al., 2025). The term “puberty” is customarily used to encompass distinct endocrinological processes that are temporally related (e.g., pubarche and axillarche as triggered by adrenal hormones; thelarche and testarche as triggered by gonadal hormones). Here we established that the start of adolescent brain structural remodeling is yet another temporally related process, regardless of whether pubertal endocrinology is its cause or merely a correlational marker. We can logically conclude that puberty does not only mark new changes to the structure of the body, but also the start of new changes to the structure of the brain.

## CRediT authorship contribution statement

**Jordan A. Chad:** Writing – original draft, Supervision, Formal analysis, Conceptualization. **Olivia C. Klukas:** Writing – original draft, Formal analysis. **Mohammad Ghasoub:** Writing – review & editing, Supervision. **Catherine Lebel:** Writing – review & editing, Supervision.

## Ethics declaration

Written informed consent to take part in the study and to publish the article has been obtained from all participants or their legal representatives. The privacy rights of participants have been observed.

This study was performed in compliance with relevant laws, regulatory frameworks and guidelines where the research took place. This study was conducted in accordance with NIMH Data Archive (NDA). This study was approved by the University of California San Diego. (Approval No. 160091)

## Funding

This work was supported by an NSERC Postdoctoral Scholarship (JAC), an O’Brien Centre Summer Studentship (OCK), a BRAIN CREATE Scholarship (MG), and an NSERC Discovery Grant (CL).

## Declaration of Competing Interest

The authors declare the following financial interests/personal relationships which may be considered as potential competing interests: Catherine Lebel is on the editorial board of Developmental Cognitive Neuroscience. She had no involvement in the peer review of this article and had no access to information regarding its peer review. Full responsibility for the editorial process for this article was delegated to another journal editor. All other authors declare that they have no known competing financial interests or personal relationships that could have appeared to influence the work reported in this paper.

## Data Availability

All data analyzed in the current study are publicly available in the NIMH Data Archive, https://nda.nih.gov/study.html?id=2147
